# 

**Published:** 1902-04-19

**Authors:** 


### NOTICE TO CORRESPONDENTS.

All MSS., Letters, Books for Review, and other matters intended for the Editor should be addressed The Editor, "The Hospital," The Hospital Building, 28 & 29 Southampton Street, Strand, W.C.

The Editor cannot undertake to return rejected MS., even when accompanied by stamped directed envelope.

**Notice to Advertisers and Subscribers.**

All Advertisements, Orders for Copies of The Hospital, and Business Communications should be addressed to **THE MANAGER, (Not to the Editor)**, The Hospital, 28 & 29 Southampton Street, Strand, London, W.C.

The Cost of Subscription, post free, is as follows.—Per week, 2½d.; per quarter, 2s. 8d.; per half-year, 5s. 3d.; per annum, 10s. 6d. Foreign:—Per annum, 15s. 2d., payable, in all cases, in advance.

**NOTICE.—Vol. XXXI. of "The Hospital" (October 1901, to March 1902), in handsome cloth binding will shortly be on sale at the office of this Journal, price, 7s. 6d. Cases for binding the Numbers contained in this Volume 1s. 6d. Index to Vol. XXXI., 2½d., post free.**

**The Monthly Part for April is now ready. Price 6d., by post 8d.**

### BOOKS RECEIVED.

Longmans, Green, and Co.

"Selected Essays and Addresses." By Sir James Paget, F.R.C.S.

P. S. King and Son.

"Educated Working Women." By Clara E. Collet, M.A.

Burlington House, Cambridge.

"University Correspondence College. The Calendar, 1901–1902."

Swan Sonnenschein and Co.

"A Text-Book of Insanity." By Charles Mercier, M.B., M.R.C.P., F.R.C.S.

"Health, Speech, and Song." A Practical Guide to Voice-Production. By Jutta Bell-Kanske.

"Small-Pox." By C. Godfrey Gümpel.

Grant Richards.

"Hospital Sketches." By Lucas Galen.

Elliot Stock.

"Woman's Best Work and Latent Capabilities." By Frederick James Grant, F.R.C.S.

J. B. Lippincott and Co.

"Photographic Atlas of the Diseases of the Skin." By George Henry Fox, A.M., M.D. Parts XIII. to XVI.

Baillière, Tindall and Cox.

"The Case for Vaccination." By G. Brown, M.R.C.S., L.R.C.P.

Young J. Pentland.

"Handbook of Obstetric Nursing." By Francis W. N. Haultain, M.D., F.R.C.P.Ed., and James Haig Ferguson, M.D., F.R.C.P.Ed., M.R.C.S.Eng. Fourth edition.

Houlston and Sons.

"'First Aid' to Injured and Sick." By J. F. Sutherland, M.D.

"Glasgow Medical Journal" Office.

"The Nitro-propiol Test for Sugar in Urine, based on 200 Observations." By Carstairs Dougla-, M.D., B Sc., &c.

Jas. Truscott and Son.

"Report of the Surgical Registrar of University College Hospital."

The Clarion Press.

"Does Municipal Management Pay?" By R. B. Suthers.

Boston City Hospital.

"Medical and Surgical Reports." Twelfth Series.

Silsbury Bros., Shanklin.

"The Child-Healer." By George H. R. Dabbs, M.D.

W. Straker.

"A Plea for Sense and Science." By John Atkinson, F.R.C.V.S.

### Scraps and Gleanings.

The report of the "Marie Celeste" Samaritan Society of the London Hospital showed that 6,286 patients had been assisted by the society during the past year; 1,513 patients who had been treated in the hospital were sent away to convalescent homes, the society making a special point of administering relief of this kind to patients of the London Hospital. The expenditure amounted to £3,812, which was an excess over income of £66.

THE Royal Asylum for the Deaf and Dumb Poor (which has institutions both at Margate and in the Old Kent Road) offers free board, clothing, and education, to the afflicted children of the poorer classes. Funds are badly needed to carry on the work. A subscription of £5 5s. entitles the subscriber to one vote at each election for the remainder of his life, while 10s. 6d. can be paid annually for every vote required.

### THE STUDENT OF MEDICINE.

### APPOINTMENTS.

**Hon. G. H. Butler**, M.R.C.S.Eng., L.R.C.P.Lond., appointed Honorary Medical Officer to the Hobart Hospital, Tasmania. **W. J. S. Bythell**, B.A., M.B., Ch.B., appointed Junior Anæsthetist to the Manchester Royal Infirmary. **Lucy B. Clapham**, M.B.Lond., appointed Obstetric Physician to the New Hospital for Women, Euston Road, and Assistant Anæsthetist to the Royal Free Hospital. **James C. Collins**, M.B., B.Ch., Trin. Coll., Dub., appointed Senior Medical Officer at the Auckland Hospital, New Zealand. **Harrie Cox**, M.B.Syd., appointed Government Medical Officer and Vaccinator at Warren, New South Wales. **T. G. Davy**, M.R.C.S.Eng., L.S.A., appointed Honorary Medical Officer to the Fremantle Hospital, West Australia. **Edward G. L. Erson**, L.R.C.P.Edin., L.M., appointed Health Officer at Peak Hill, Western Australia. **N. Bishop Harman**, M.A., M.B.Cantab., F.R.C.S. Eng., appointed Ophthalmic Surgeon to the Belgrave Hospital for Children. **Charles J. Harriett**, M.D.Lond., M.R.C.S., L.R.C.P., appointed Honorary Assistant Visiting Surgeon to the Royal Sea-Bathing Hospital, Margate. **R. E. Hodgson**, M.R.C.S., L.R.C.P., appointed Senior Resident Surgeon to the Royal Sea-Bathing Hospital, Margate. **W. M. Hunter**, M.B., B.Ch.R.U.I., appointed Certifying Factory Surgeon for the Crumlin District of Antrim. **John Larwill**, L.R.C.P., L.R.C.S.Edin., appointed Medical Officer at Boonah, Queensland. **James Thomas Mitchell**, M.D., Ch.M.Aberd., M.R.C.S.Eng., appointed Resident Medical Officer to the Ballarat Benevolent Asylum and Lying-in Home. **Hubert C. Phillips**, M.R.C.S.Eng., M.&L.S.A.Lond., appointed Anæsthetist to the Female Lock Hospital, Harrow Road. **W. James Reiach**, M.B., C.M.Edin., appointed Government Medical Officer and Vaccinator at Molong, New South Wales. **W. H. Reid**, M.B., Ch.M.Syd., appointed Junior Medical Officer in the Department of Lunacy of New South Wales. **Leonard Douglas Saunders**, M.R.C.S.Eng., L.R.C.P.Lond., appointed House Surgeon to the Hastings, St. Leonards, and East Sussex Hospital, Hastings. **Edwin Fraser Seabrook**, M.D., appointed Health Officer under the Quarantine Acts for South Australia. **L. L. Seabrook**, Jun., M.B., Ch.B.Adelaide, appointed Resident Medical Officer of Broken Hill Hospital, New South Wales. **Grace Soltau**, L.R.C.P.&S.Edin., appointed House Surgeon to the Belgrave Hospital for Children. **John W. Spence**, L.R.C.P., L.R.C.S.Edin., appointed Non-Resident House-Physician and Surgeon to the Deaconess Hospital, Edinburgh. **Thomas Grainger Stewart**, M.B., Ch.B.Edin., appointed Junior House Physician to the National Hospital for the Paralysed and Epileptic. **Dawson Turner**, B.A., M.D.Edin., appointed Examiner in Experimental Physics to Edinburgh University. **E. R. Turner**, M.B., C.M.Aberd., appointed Medical Officer and Vaccinator for the Parish of Kinellar. **Ethel M. Vernon**, M.D., B.S.Lond., L.S.A., appointed Pathologist to the New Hospital for Women, Euston Road. **J. Wheatley**, M.D.Lond., appointed Medical Officer of the County of Shropshire. **Margaret White**, M.B., Ch.M.Syd., appointed House-Surgeon to the Children's Hospital, Adelaide, South Australia. **Oliver K. Williamson**, M.A., M.B., B.C.Cantab., M.R.C.P.Lond., M.R.C.S.Eng., appointed Medical Registrar to the Middlesex Hospital. **Arthur S. Wohlmann**, M.D., B.S.Lond., appointed Official Balneologist to the Government of New Zealand. **James Francis Wolfe**, M.B., C.M.Edin., appointed Medical Officer to the Heavitree (Exeter) Isolation Hospital. **Charles Yeoman**, M.B., appointed Medical Officer for the Hollington District of the Battle Union.

